# A theoretical model of dietary lipid variance as the origin of primary ciliary dysfunction in preeclampsia

**DOI:** 10.3389/fmolb.2023.1173030

**Published:** 2023-05-05

**Authors:** Nancy R. Hart

**Affiliations:** PeaceHealth, Bellingham, WA, United States

**Keywords:** primary cilia, preeclampsia, membrane signaling, Hedgehog, lipidomics, accessible cholesterol, polyunsaturated fat, lipid rafts

## Abstract

Serving as the cell’s key interface in communicating with the outside world, primary cilia have emerged as an area of multidisciplinary research interest over the last 2 decades. Although the term “ciliopathy” was first used to describe abnormal cilia caused by gene mutations, recent studies focus on abnormalities of cilia that are found in diseases without clear genetic antecedents, such as obesity, diabetes, cancer, and cardiovascular disease. Preeclampsia, a hypertensive disease of pregnancy, is intensely studied as a model for cardiovascular disease partially due to many shared pathophysiologic elements, but also because changes that develop over decades in cardiovascular disease arise in days with preeclampsia yet resolve rapidly after delivery, thus providing a time-lapse view of the development of cardiovascular pathology. As with genetic primary ciliopathies, preeclampsia affects multiple organ systems. While aspirin delays the onset of preeclampsia, there is no cure other than delivery. The primary etiology of preeclampsia is unknown; however, recent reviews emphasize the fundamental role of abnormal placentation. During normal embryonic development, trophoblastic cells, which arise from the outer layer of the 4-day-old blastocyst, invade the maternal endometrium and establish extensive placental vascular connections between mother and fetus. In primary cilia of trophoblasts, Hedgehog and Wnt/catenin signaling operate upstream of vascular endothelial growth factor to advance placental angiogenesis in a process that is promoted by accessible membrane cholesterol. In preeclampsia, impaired proangiogenic signaling combined with an increase in apoptotic signaling results in shallow invasion and inadequate placental function. Recent studies show primary cilia in preeclampsia to be fewer in number and shortened with functional signaling abnormalities. Presented here is a model that integrates preeclampsia lipidomics and physiology with the molecular mechanisms of liquid–liquid phase separation in model membrane studies and the known changes in human dietary lipids over the last century to explain how changes in dietary lipids might reduce accessible membrane cholesterol and give rise to shortened cilia and defects in angiogenic signaling, which underlie placental dysfunction of preeclampsia. This model offers a possible mechanism for non-genetic dysfunction in cilia and proposes a proof-of-concept study to treat preeclampsia with dietary lipids.

## 1 Introduction

Many eukaryotic cells contain cilia, antenna-like organelles projecting from the cell surface, composed of microtubules organized from a centriole-derived basilar body. Cilia are externally bounded by membrane that differs in composition from the surrounding plasma membrane and unbound by a membrane at their basal interface with cellular cytoplasm. Motile cilia such as flagella are well-known. The less familiar primary cilia, with differing arrangements of microtubules, are now a focus of multidisciplinary research (Arora et al., 2023), with primary cilia emerging as centers for myriad signaling events, including Hedgehog (Hh), G-protein coupled receptors, Wnt, and tyrosine kinase (Anvarian et al., 2019), among others (Wheway et al., 2018). Serving as sensing centers, cilia coordinate communication to provide cellular responses to extracellular stimuli (Pala et al., 2017). Classically, ciliopathies originate in gene mutations that code for proteins that are important in the formation or function of cilia (Waters and Beales, 2011; Wheway et al., 2019; Focșa et al., 2021). However, recently, cilia have been implicated in conditions without clear genetic antecedents, such as obesity (Wu et al., 2023), diabetes (Liu et al., 2022; Melena and Hughes, 2022), neurodegenerative diseases (Dhekne et al., 2018; Ki et al., 2021; Nguyen et al., 2021), cancer (Kimura et al., 2023), psychiatric disorders (Alhassen et al., 2021), autoimmune disorders (Martínez-Hernández et al., 2019; Chen et al., 2021; Nishimura et al., 2021), and cardiovascular diseases (Pala et al., 2018; Wang et al., 2021) including preeclampsia (Wang et al., 2019a; Ritter et al., 2020; Romberg et al., 2022).

As with genetic ciliopathies, in preeclampsia, multiple maternal organ systems may be affected with clinical symptoms ranging from seizures, stroke, anasarca, and blindness to liver and kidney failures. Although women who develop preeclampsia may appear normal at the onset of pregnancy, epidemiologic studies show an increased risk for preeclampsia in those with dyslipidemia (Enquobahrie et al., 2004), elevated trans fatty acids (TUFA) (Williams et al., 1998), or reduced omega-3 fatty acids (Williams et al., 1995) in early pregnancy. Clinically, preeclampsia is a disease of the maternal vascular endothelium with impaired vasodilation (Rezeck Nunes et al., 2022) and increased vasoconstriction (Leal et al., 2022), resulting in hypertension, increased capillary basement membrane permeability to water (Wang et al., 2007) with marked edema, and impaired renal resorption of protein (Genest et al., 2021), resulting in proteinuria (Rana et al., 2019). Pathologically, preeclampsia resembles chronic hypertension with lipid laden macrophages, atherosclerotic streaks, reduced renal perfusion, and hypercoagulability. The fetus generally does not show these pathological changes. Not only is preeclampsia one of the leading causes of maternal morbidity and mortality worldwide (Ghulmiyyah and Sibai, 2012), but after a pregnancy affected by preeclampsia, for the mother, there is an eight-fold increased risk for death from CVD (Brown et al., 2013), and the fetus experiences an increased lifetime risk of hypertension, obesity, and neurologic diseases (Bakrania et al., 2020). In an analysis of over fifty million live births in the United States from 2007 to 2019, the incidence of new-inset hypertensive disorders doubled (Cameron et al., 2022), continuing a documented trend of increasing incidence over the previous 2 decades. (Wallis et al., 2008). Although metabolomic studies show no clear genetic antecedents, major alterations in lipid metabolism have caused some researchers to describe preeclampsia as a lipid storage disease (Melland-Smith et al., 2015) with consistent findings of sterol, phospholipid, and sphingolipid metabolism as major discriminatory pathways (Brown et al., 2016; Lee et al., 2020; He et al., 2021; Li et al., 2021). The cause of preeclampsia is unknown, with the only cure being the delivery of the fetus, often quite prematurely.

In the last 20 years, models of preeclampsia etiology have focused on the primacy of poor placental development due to increased apoptosis and impaired angiogenesis (Rana et al., 2019), with Hedgehog (Hh) (Lawson et al., 2002), VEGF (Wang et al., 2017), and Wnt signaling (Wang et al., 2018) all downregulated in preeclampsia (Huang et al., 2021). Recent findings reveal that Hh signaling, which is located upstream of VEGF signaling to promote angiogenesis, is positively regulated by accessible membrane cholesterol, that is, cholesterol that is neither esterified nor sequestered by sphingomyelin (Radhakrishnan et al., 2020; Kinnebrew et al., 2021). In the Wnt/catenin pathway, cholesterol acts as a switch, advancing the proangiogenic canonical pathway over the non-canonical apoptosis-promoting pathway (Sheng et al., 2014). In preeclampsia, trophoblastic primary cilia, the loci of proangiogenic signaling, are shortened and fewer in number (Wang et al., 2019b) with diminished Hedgehog signaling (Ritter, 2020), causing functional impairment in the developing placenta. In other species, similar abnormalities in ciliogenesis and function occur with membrane cholesterol depletion (Maerz et al., 2019). In preeclampsia, downregulation of endothelial nitric oxide synthase to form the vasodilator nitric oxide occurs when caveolar cholesterol is depleted (Levitan and Shentu, 2011). Each of these pathways points to a deficiency of cell membrane cholesterol in preeclampsia. However, in preeclampsia, as in CVD, systemic cholesterol seems to be in excess, with cholesterol laden macrophages and elevated total serum cholesterol (Spracklen et al., 2014). These findings present a paradox: how is it possible for membranes to lack accessible cholesterol in the presence of excessive systemic cholesterol?

## 2 Hypothesis

This perspective presents a theoretical model of the mechanism in preeclampsia by which transplacental movement of cholesterol from the maternal to the fetal system and increased sequestration of cholesterol due to variations in dietary lipids may cause an acute deficiency in accessible membrane cholesterol. This cholesterol deficit results in abnormalities in the formation and function of primary cilia, impaired placental angiogenesis, and clinical preeclampsia. To test this hypothesis, treatment using lipid therapy is proposed for those diagnosed with early preterm preeclampsia to reverse the pathologic events that lead to fulminant hypertension, preeclampsia, and preterm delivery.

## 3 Changes in dietary lipids

Over the last century, humans in the United States have undergone a widespread, uncontrolled experiment by markedly increasing the quantity of polyunsaturated fatty acids (PUFAs) in their diets. From 1909 until 2017, the United States Department of Agriculture Economic Research Service maintained data on yearly food consumption in the United States. These data show *per capita* consumption of butter, lard, red meat, grains, eggs, and milk products decreased, while salad oil, cooking oil, shortening, poultry, and fish intake increased. Over the last century, there has been a *per capita* reduction in yearly dietary intake of saturated fat from 28 pounds in 1912 to 13.4 pounds in 2011, while PUFA consumption from vegetable oils increased from 11.3 to 64.5 pounds. During this time, total dietary fat has increased from 41 to 79 pounds *per capita* per year (Gerrior et al., 2004), an increase almost totally attributable to increased polyunsaturated vegetable oils (USDA/ERS U.S, 2021).

Several sources suggest that human beings evolved on a diet with a ratio of omega-6:3 PUFA of about 1:1, whereas in Western diets, the ratio averages 16:1, with ratios in urban areas of India ranging between 38:1 and 50:1 (Cordain et al., 2002; Simopoulos, 2002). For this reason, Western diets are considered relatively deficient in omega-3 fatty acids (Simopoulos and Cleland, 2003). Considered essential dietary nutrients, humans are not able to synthesize omega-6 and 3 fatty acids *de novo* or interconvert them. Because of the competition for desaturase enzymes, it is the balance or ratio of the two fatty acids that determines the relative abundance of downstream metabolites (Bézard et al., 1994). Oxidation of omega-3 and 6 PUFAs forms potent signaling eicosanoids such as prostaglandins and thromboxanes. Generally, eicosanoids derived from omega-6 and 3 PUFA have opposing effects: omega-6-derived eicosanoids increase and omega-3-derived eicosanoids decrease thrombosis, inflammation, and vasoconstriction (Schmitz and Ecker, 2008).

Worldwide, the intake of TUFAs in the form of hydrogenated or partially hydrogenated vegetable oils rose markedly over the 20th century from an optimum of less than 1% of total energy intake to a high of 6.5% in some countries (Craig-Schmidt, 2006; Wanders et al., 2017). In 1990, the estimated daily TUFA intake in the United States was 11–27.6 g per person per day, with a total of 66 g of fat daily in a 2000-calorie diet (Enig et al., 1990). Although estimated dietary TUFA has plateaued or fallen over the last 20 years with the World Health Organization’s initiative to limit the production of hydrogenated vegetable oils (Bösch et al., 2021), TUFA forms in cooking oil at a rate of roughly 3.5 g of TUFA per 100 g of oil with each cooking episode (Bhardwaj et al., 2016). Deep fryers that reuse cooking oil and the practice of incorporating spent cooking oil into livestock feeds ensure an ongoing supply of trans fats in human foods (Arowolo et al., 2020).

Archeologic studies estimate the average daily cholesterol intake of our pre-agricultural ancestors at 480–880 mg/day (Eaton and Konner, 1985; Cordain et al., 2000; Konner and Eaton, 2010; Kuipers et al., 2010), compared to 293 mg/day in the United States in 2014 (Xu et al., 2018). With its first dietary guidelines in 1980, the USDA recommended limiting dietary cholesterol to less than 300 mg daily to promote health and reduce CVD. The 2010 guidelines introduced the “Dietary Approaches to Stop Hypertension Diet,” which recommended no more than 136 g of cholesterol daily and limits on total and saturated fats for those with CVD (USDA, 2010). In a marked departure from previous guidelines, in 2015, the recommendation to limit daily cholesterol to less than 300 mg daily was eliminated (U.S. Department of Health and Human Services and U.S. Department of Agriculture, 2015). While a vigorous discussion regarding optimal daily intakes of cholesterol and saturated and unsaturated fats continues to be unresolved, there is little disagreement that oxysterols, which form endogenously or when cholesterol is heated in the presence of oxygen, are implicated in diseases in which inflammation, oxidation, and increased autophagy play a pathophysiologic role such as neurodegenerative diseases, CVD, and cancers (Poli et al., 2013). Substantial levels of oxysterols are present in commercial food products and foods cooked at high temperatures (Schroepfer, 2000). Because levels of oxysterol formation vary significantly by preparation technique, it is difficult to estimate average dietary consumption.

In summary, compared to diets of our genetic ancestors, 21st-century diets in the USA contain about six times as much PUFAs with a significantly elevated omega 6:3 ratio, reduced saturated fat, about half of the cholesterol content, and markedly more TUFAs.

## 4 Phase separation of lipids

### 4.1 Phase separation of *in vitro* lipid mixtures

**FIGURE 1:**
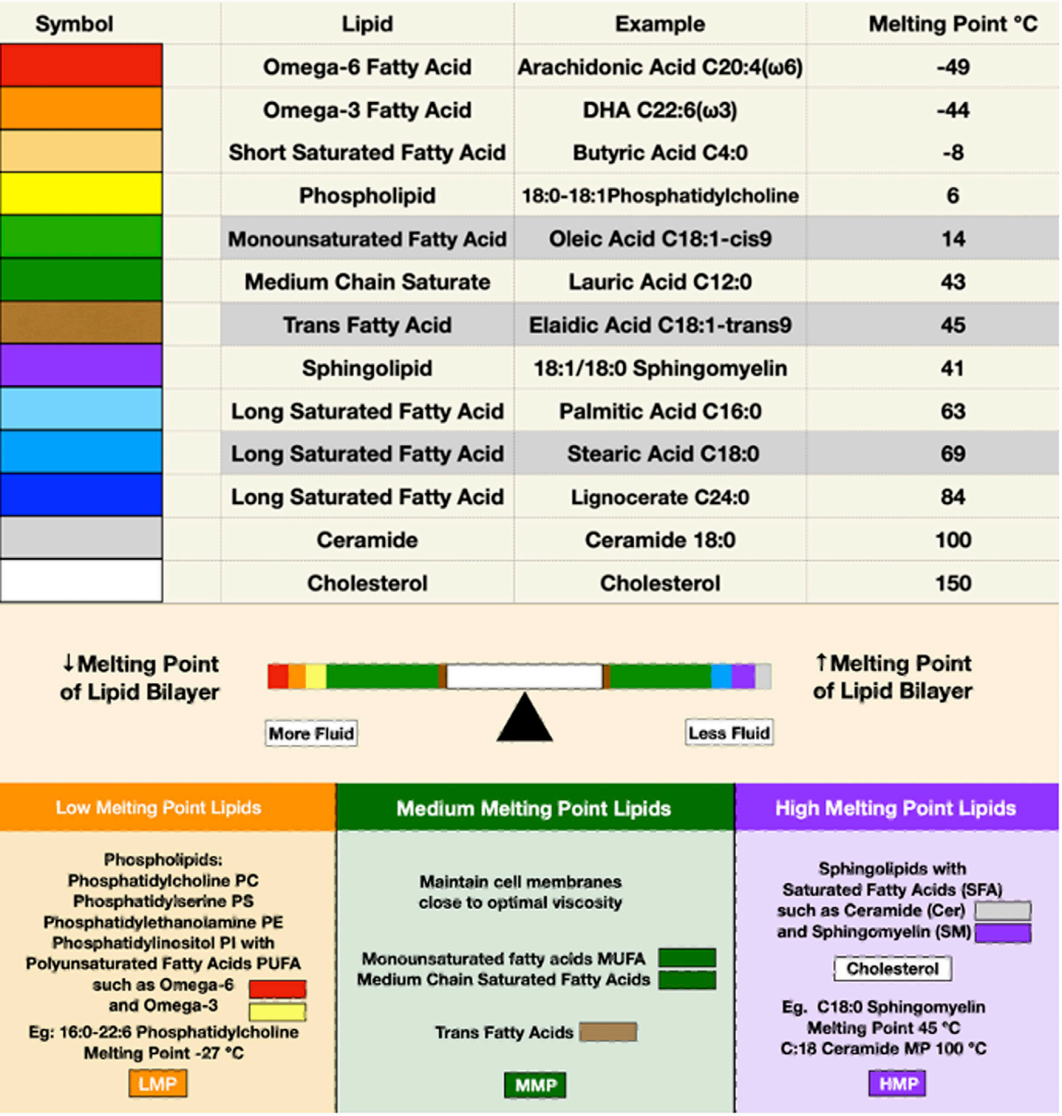
The upper panel depicts the melting points of some representative membrane lipids. Red, orange, and yellow represent lipids with low melting points; green and brown for moderate melting points; and purple, blue, and gray for high melting points. The balance bar shows the effects of lipids on membrane fluidity, with low melting point lipids increasing and high melting point lipids reducing membrane bilayer fluidity. Cholesterol, depicted in white, is in the middle of the balance beam because it maintains optimal viscosity over a wide range of conditions despite its high melting point.

**FIGURE 2:**
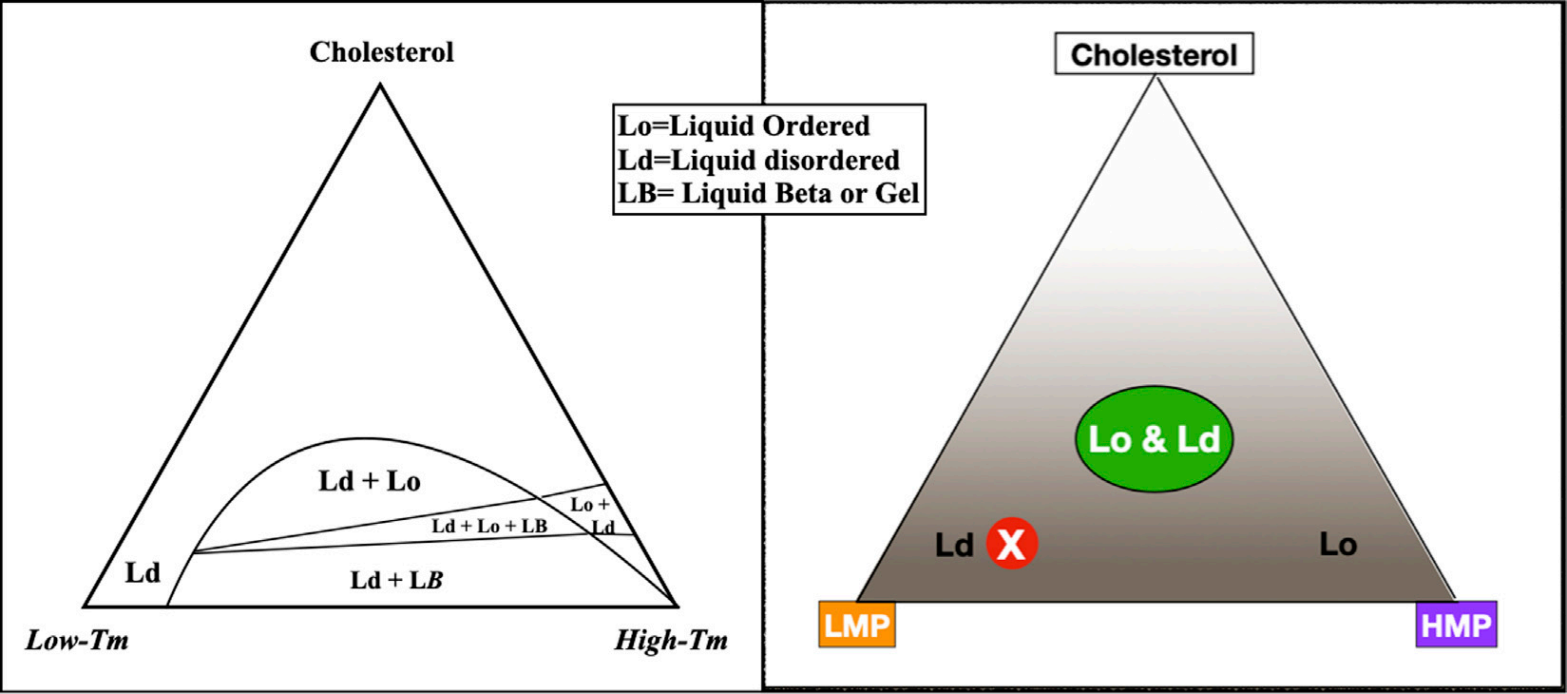
To describe the behavior of varying lipid mixtures in phase separation, a three-sided graph may be used, such as the figure on the left, simplified to the figure on the right. In this graph, the three corners represent 100% molar concentrations of constituent lipids; moving away from the corners, concentrations decrease. For example, the peak of the triangle is an area of high cholesterol; at the base of the triangle, cholesterol concentration is low. Within the triangle, each point represents a unique combination of three lipids. The concentration of any component in the mixture may be seen by extending a line perpendicularly from any point on the graph to the corresponding side of the triangle. For example, a mixture with mostly low melting point lipid, a small amount of high melting point lipid, and low cholesterol plots to the red circled X, in an area of liquid disorder. The area labeled Lo & Ld with a green oval on the right represents mixtures where liquid-ordered and -disordered phases coexist, roughly corresponding to lipid rafts in living cell membranes.

In the lab, a bilayer membrane forms spontaneously if phospholipids are mixed in an aqueous solution. Varying the saturation and chain lengths of fatty acids affects the membrane melting point, with longer chains and fewer double bonds raising the melting point and shorter chains and more double bonds lowering the melting point. Although cholesterol has a high melting point, it serves as a membrane plasticizer, keeping membranes at optimal viscosity over a wide range of conditions (Figure 1). A mixture of a low melting point lipid such as polyunsaturated phosphatidylcholine, a high melting point lipid such as saturated sphingomyelin (SM), and cholesterol spontaneously forms a bilayer membrane with areas of differing liquid order or phases. The behavior of different lipid mixtures in phase separation has been mapped and shown as ternary lipid mixture phase diagrams, with some mixtures allowing the simultaneous existence of both liquid-ordered and liquid-disordered phases (Figure 2).

**FIGURE 3:**
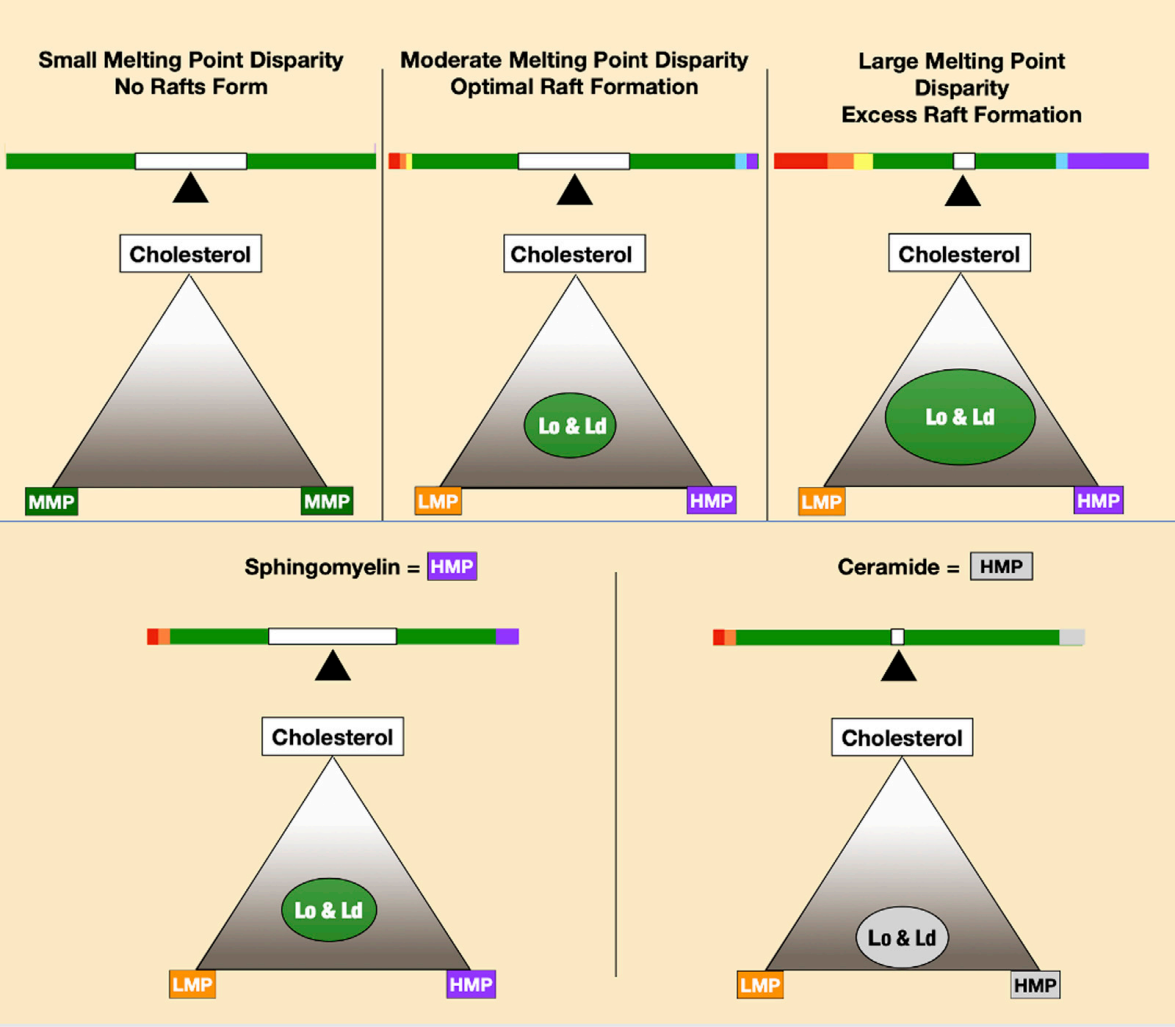
Increasing melting point disparity causes increasing phase separation of lipid mixtures, as seen in the top three panels. The two lower panels depict ceramide-forming rafts at lower cholesterol concentrations than sphingomyelin.

The basis of phase separation is the tendency of saturated sphingolipids and phospholipids to associate with cholesterol and dissociate from PUFAs. Disordered phases enriched in low melting point lipids provide bilayer fluidity that contrasts with tightly packed high melting point lipids of the ordered phase. In model membrane systems, the melting point disparity of constituent lipids is a determinant of lipid raft formation and stability (Levental et al., 2016; Levental et al., 2020a), meaning lipid polyunsaturation is a major driving force for liquid–liquid phase separation (Lin et al., 2016). A greater disparity in melting points of membrane lipids favors greater phase separation (Figure 3).

### 4.2 Lipid rafts *in vivo*

Membrane rafts are defined as small, dynamic, heterogeneous, and sterol-, and sphingolipid-enriched membrane domains that compartmentalize cell membrane processes and serve as organizing centers for a complex network of signaling activity (Simons and Toomre, 2000). Most raft-associated pathologies, including hypertension and atherosclerosis, are caused by “excessive” lipid rafts, that is, increased raft abundance or stability (Sviridov et al., 2020). Although the primacy of proteins versus lipids in organizing membrane rafts *in vivo* is debated, the liquid-ordered phase of *in vitro* model membranes roughly corresponds to membrane rafts in living cells (Korade and Kenworthy, 2008).

**FIGURE 4:**
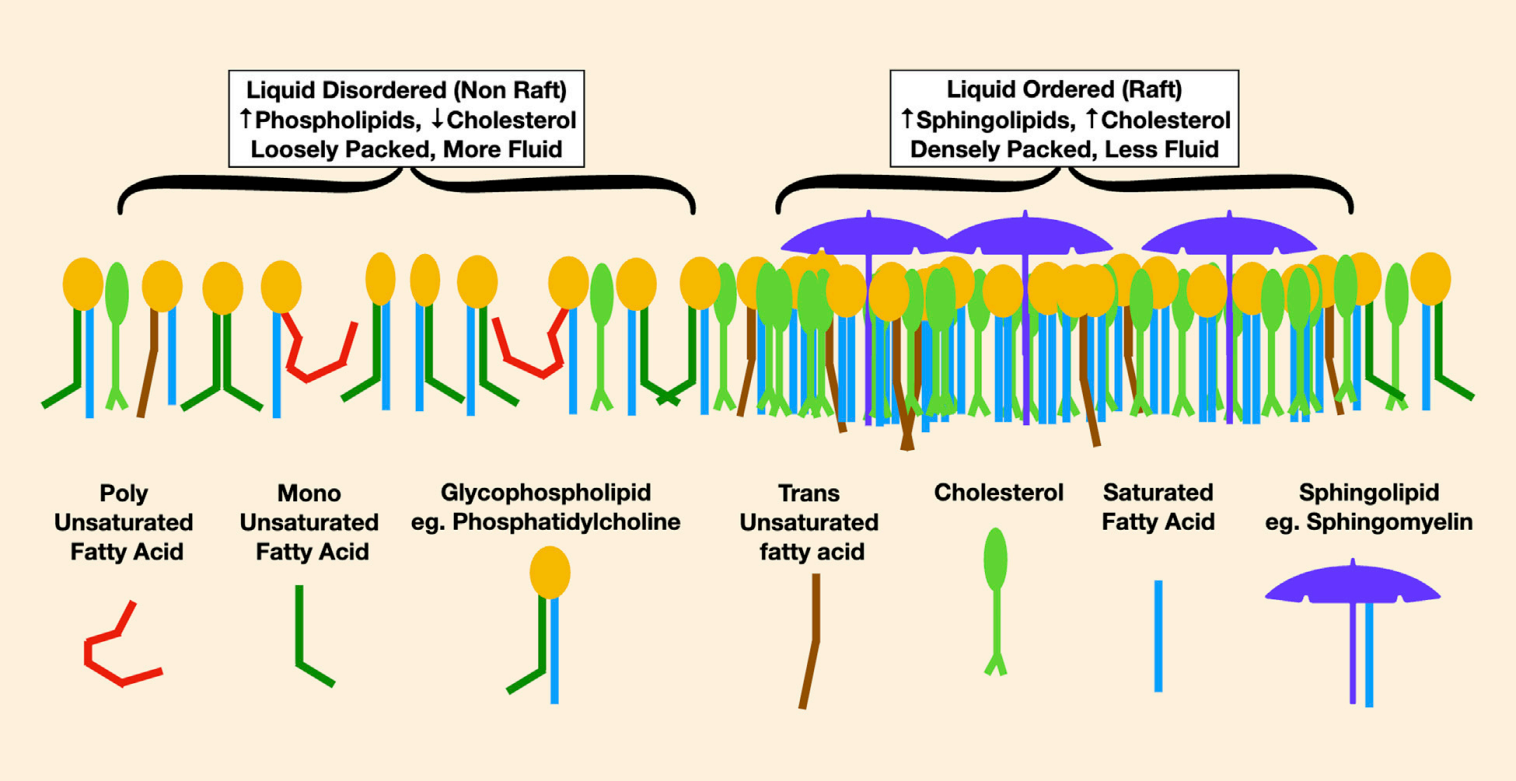
This is a representation of lipids in the outer leaflet of a cell membrane. On the left, the liquid-disordered areas are more loosely packed with less cholesterol. In red, the effect of a very low melting point polyunsaturated fatty acid in reducing packing density is noticable, causing increased membrane fluidity. On the right are densely packed rafts enriched in high melting point lipids such as long-chain saturated sphingomyelin, pictured as an umbrella, and lots of cholesterol.

Most of our understanding of lipid rafts arises from studies of the outer cell membrane leaflet. In this lipid monolayer, liquid-disordered areas are enriched in lower melting point phospholipids such as phosphatidylcholine, in which an unsaturated fatty acid reduces cholesterol affinity. Liquid-ordered areas are enriched in densely packed sphingolipids esterified with saturated fatty acids, which have high cholesterol affinity. Thus, liquid-ordered areas may be imagined as cholesterol- and sphingolipid-rich rafts floating in a liquid-disordered sea (Figure 4).

In cell membranes, the properties of lipid rafts may be modulated by changes in lipid availability. Depletion of cholesterol from a membrane using cyclodextrin is a well-known method to break up membrane rafts (Zhuang et al., 2005). Enrichment of membranes with ceramide alters raft structure and causes displacement of cholesterol from association with sphingomyelin (Megha and London, 2004). Oxysterols in low-density lipoprotein (LDL) deplete caveolar cholesterol and induce the formation of ceramide-rich rafts, while monounsaturated fatty acids inhibit raft formation and PUFAs stabilize rafts (Sviridov, 2020). Thus, membrane lipid composition is critical to the formation and function of membrane rafts. In humans, there is a vast metabolism to alter fatty acids with enzymes to saturate, desaturate, cleave, and elongate; however, most fatty acids are initially rapidly incorporated into cell membranes unchanged from the form in which they are absorbed from the gastrointestinal tract (Levental et al., 2020b). Therefore, dietary lipids may have a major effect on the composition and function of endothelial membranes and rafts.

Compared to their cis analogs, TUFAs require eight times as much cholesterol to be incorporated into a cell membrane (Niu et al., 2005; Oteng et al., 2019). TUFAs may bind to either sphingolipids or phospholipids but tend to associate in rafts due to their high cholesterol affinity, thereby increasing raft formation.

### 4.3 Ceramides, rafts, and accessible cholesterol

Some cholesterol in plasma membranes is considered “free” or “accessible” and is available for signaling and providing feedback inhibition of cholesterol synthesis, thereby protecting against cholesterol overaccumulation in the plasma membrane. However, cholesterol associated with sphingomyelin, long-chain saturated fatty acids, TUFAs in lipid rafts, or esterified to fatty acids, is not accessible (Das et al., 2014). Lipidomic studies of preeclampsia show disordered sphingolipid metabolism with increased ceramides in those affected (Korkes et al., 2014; Dobierzewska et al., 2017; He et al., 2021) Allowing liquid-ordered phase separation at lower cholesterol concentrations than sphingomyelin, ceramides displace cholesterol from sphingomyelin, creating accessible cholesterol (Megha and London, 2004). Heat stress, which increases membrane fluidity, also increases *de novo* ceramide synthesis (Chang et al., 1995). In lipid bilayers, ceramide increases permeability to water and trans-bilayer lipid movements (flip-flop) (Goñi et al., 2014).

## 5 Homeoviscosity and dietary lipids *in vivo*

In 1974, Sinenski coined the phrase “homeoviscous adaptation” to describe the process that regulates the viscosity of membrane lipids in *Escherichia coli*, noting that as temperature increases, cells increase the content of long-chain saturated fatty acids, thus maintaining a constant viscosity (Sinensky, 1974). Both plants and animals adjust the balance of low and high melting point lipids in tissues to maintain homeoviscosity. When humans eat other organisms as food, the intake of fatty acids may vary depending on environmental conditions. For example, the total omega-3 fatty acid content of grass-fed beef is significantly greater in the spring than in the fall (Jain et al., 2020). For optimal function, organisms must maintain tight parameters on membrane viscosity.

One confusing aspect in considering homeoviscosity and dietary lipids is that PUFAs lower the membrane melting point and increase lipid bilayer fluidity. However, preeclampsia and other CVDs are characterized by increased cellular and arterial stiffness as measured by atomic force microscopy (Lee et al., 2011), RBC deformability (Schauf et al., 2002), and pulse wave velocity (Hausvater et al., 2012). The key to this paradox is that increases in lipid bilayer fluidity cause a compensatory increase in cytoskeletal elements, resulting in stiffer cells. Bilayer fluidity is, thus, directly related to cell stiffness, with more fluid cell membrane bilayers being associated with stiffer cells (Shentu et al., 2012; Efremov et al., 2019; LührJennifer et al., 2020).

## 6 The cholesterol paradox

In answer to our earlier question, “How is it possible for membranes to be deficient in accessible cholesterol in the presence of elevated systemic cholesterol?” we might reason as follows: high dietary levels of PUFAs, when incorporated into cell membranes, increase membrane fluidity. To maintain homeoviscosity in this setting, cells must add higher melting point lipids, such as long-chain saturated fatty acids, either from dietary sources or *de novo* synthesis. This results in a high melting point disparity of membrane lipids and increased membrane rafts, which sequester cholesterol. Increased levels of dietary TUFAs, which have a high cholesterol affinity compared to their cis analogs, also increase cholesterol sequestration and raft formation. Highly unsaturated fatty acids are quite vulnerable to free radical damage and oxidation, causing damaged fatty acids that must be esterified to cholesterol for removal from a cell, further reducing accessible cholesterol. By these mechanisms, high levels of dietary PUFAs and TUFAs might directly cause both the increased membrane rafts, which are characteristic of CVD, and a deficiency in accessible membrane cholesterol. Another major mechanism that reduces accessible membrane cholesterol in the maternal endothelium is the transfer of cholesterol from the maternal circulation across the placenta to the fetus (Woollett, 2011; Woollett et al., 2023). By all these mechanisms, an acute reduction in accessible membrane cholesterol provides a coherent explanation for preeclampsia’s reduction in endothelial nitric oxide synthase activity, which is inactivated by the depletion of caveolar cholesterol and the impairment of cholesterol-dependent Hh, Wnt, and VEGF signaling. With the delivery of the placenta and fetus, the drain on maternal cholesterol ends, explaining the very rapid resolution of preeclampsia after parturition.

In response to increased cellular demand for cholesterol caused by sequestration, cholesterol synthesis may be increased (Lee et al., 2019) with increased sterol regulatory element binding protein (SREBP) -2 signaling (Vásárhelyi et al., 2006; Jianhua et al., 2016), increased hepatic synthesis and export of cholesterol in LDL (Chen et al., 2022), increased LDL receptors to transport cholesterol into peripheral cells, reduced ABCA1 activity transporting cholesterol out of cells (Körner et al., 2012; Liu et al., 2014), and reduced high-density lipoprotein (HDL) to remove excess cholesterol, changes found in both CVD and preeclampsia. Thus, the dyslipidemia, increased ceramides, reduced Hh and Wnt signaling, and abnormal structure of cilia that are found in preeclampsia may be a direct consequence of the high unsaturation index, reduced cholesterol, and increased TUFAs of modern diets.

## 7 Reduction of cilia in preeclampsia

Section 5 notes that increased membrane PUFAs, which reduce bilayer viscosity, cause an increase in cellular stiffness through increased cytoskeletal elements. Although the molecular pathways responsible for the formation and resorption of cilia are still being developed, it is known that actin polymerization creates a physical barrier to the assembly of cilia and promotes the disassembly of cilia, while inhibition of actin polymerization promotes ciliogenesis (Smith et al., 2020). In this way, elevated membrane PUFAs might reduce cilia formation. In a 2019 study, Mearz et al. show that reducing cholesterol by treating zebrafish embryos with statin drugs or cyclodextrin results in phenotypes like those that arise from defects in cilia, which are mitigated by supplying cholesterol. Cholesterol depletion also causes deficiencies in ciliary signaling. The phosphoinositide Pi(4,5) P_2_, which functions as an entrance guide at the base of the cilium and relies on Golgi sterols for formation, is reduced with cholesterol depletion. These authors surmise that cholesterol depletion is likely to disrupt ciliogenesis by altering phosphoinositide levels at the cilium (Maerz, 2019). By these mechanisms, increased dietary PUFAs and reduced membrane cholesterol might account for the reduction in the number, size, and abnormal signaling of primary cilia in preeclampsia.

## 8 Inflammation, thrombosis, and oxidative stress in preeclampsia pathology

Recently, placental ischemia has been emphasized as the origin of preeclampsia’s characteristic thrombosis and inflammation. In the 1980s, an abnormal thromboxane-to-prostacyclin ratio in maternal blood, with increased omega-6-derived inflammatory thromboxane and reduced omega-3-derived antithrombotic and vasodilating prostacyclin, was proposed as the primary cause of preeclampsia (Sibai et al., 1994). Presently, the relative importance of placental ischemia versus an altered maternal omega 6:3 ratio in creating preeclampsia’s proinflammatory and prothrombotic environment is open to discussion.

PUFAs oxidize markedly more readily than saturated or monounsaturated fatty acids, releasing free radicals that may propagate further oxidation (Halliwell, 1991). By absorbing free radicals without propagation, cholesterol (Girao et al., 1999) and other antioxidants quench this chain reaction. Oxysterols, whether formed endogenously by cholesterol oxidation or enzymatically or obtained exogenously from the diet, are increased in preeclampsia (Moon et al., 2014). Oxysterols are incorporated into cell membranes, but their properties differ from those of cholesterol (Olżyńska et al., 2020), as oxysterol-containing membranes *in vitro* are thinner with less lateral pressure (Itri et al., 2014; Siani et al., 2016) and increased water permeability (Lis et al., 2011), perhaps partially explaining the profound edema that often accompanies preeclampsia. Oxidized LDL, which transports oxidized cholesterol, increases endothelial stiffness, an impact that is fully reversed by supplying cells with a surplus of cholesterol (Shentu et al., 2010). Oxysterols also inhibit trophoblastic invasion of the endometrium (Pavan et al., 2004).

The changes in membrane properties caused by oxysterols might also explain increased angiotensin 1 receptor (AT1R) sensitivity to angiotensin II, which plays a central role in mediating the intense vasospasm of preeclampsia. Mutations causing relaxation in the intramembranous portion of AT1R cause the molecule to relax in the “on” position, causing vasoconstriction, increased blood pressure, decreased renal blood flow, and other AT1R signaling effects (Cotecchia et al., 1990; Unal and Karnik, 2014). Although a modest increase in mutated alleles of AT1R has been described in preeclampsia, in most cases, abnormal alleles of AT1R are not detected (Seremak-Mrozikiewicz et al., 2005). A possible mechanism of preeclampsia’s increased AT1R sensitivity is the altered membrane lipid environment of increased oxysterols, and reduced lateral membrane pressure allows relaxation of the intramembranous portion of AT1R, similarly to the conformational change that activates the receptor with a genetic mutation, thus allowing the molecule to relax in the “on” position.

**FIGURE 5:**
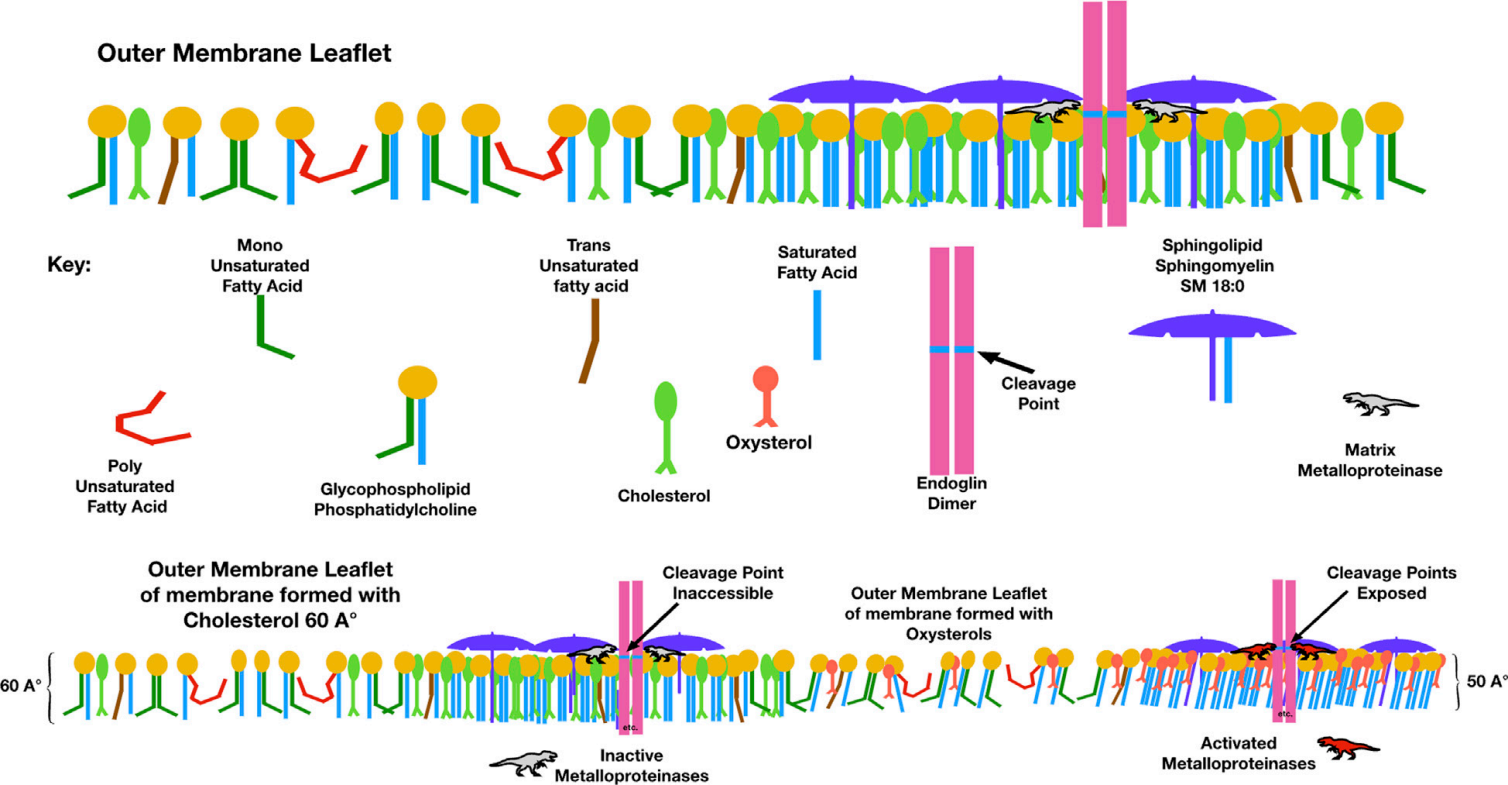
Compared to membranes formed with cholesterol, those with oxysterols are thinner and have lower lateral pressure. This figure illustrates a possible mechanism by which the elevated membrane oxysterols in preeclampsia create soluble endoglin, which, when released into the maternal circulation, binds with TGF-β and blocks its action. The point where matrix metalloproteinase-14 cleaves endoglin to release its extra-membranous soluble portion is normally sequestered within the hydrophobic plasma membrane. With high levels of oxysterols and thinner membranes, this cleavage point may be exposed.

Endoglin, a membrane receptor necessary for transforming growth factor-β (TGF-β) signaling, is essential during angiogenesis. Cleavage of endoglin by matrix metalloproteinase-14 creates a soluble receptor (sEng), which is elevated in the sera of people with preeclampsia. sEng binds TGF-β and impairs functions such as endothelial nitric oxide synthesis, vasodilation, modulation of permeability, regulation of immune homeostasis, trophoblastic invasion, and differentiation (Venkatesha et al., 2006). Membrane raft domains negatively regulate TGF-β signaling, and aspirin, which disperses membrane rafts, induces TGF-*β* signaling. The cleavage of endoglin to form sEng is upregulated in the presence of oxysterols (Valbuena-Diez et al., 2012). When incorporated into maternal endothelial membranes, perhaps oxysterol-induced membrane thinning causes a hydrophobic mismatch, exposing the cleavage site for matrix metalloproteinases to release soluble endoglin into the circulation (Figure 5).

Similarly, a soluble form of the VEGF receptor, sFlt, blocks VEGF, placental growth factor (PlGF) activity, and placental development. Maternal serum sFlt levels are increased in patients with preeclampsia, fall after delivery and are associated with reduced levels of free VEGF and PlGF. When administered to pregnant rats, sFlts induce hypertension, proteinuria, and glomerular endotheliosis in a pattern resembling human preeclampsia (Maynard et al., 2003). Acid sphingomyelinase, which converts sphingomyelin into ceramide, thereby increasing cholesterol availability, is increased in those who develop preeclampsia and is associated with increased sFlt production (Rodríguez-Sureda et al., 2016). Pravastatin, a drug developed to treat dyslipidemia by inhibiting HMG CoA reductase, the rate limiting step in cholesterol synthesis, reduces sFlts and soluble endoglin with *in vitro* treatment of human cells (Brownfoot et al., 2015). However, treatment with pravastatin increases both systemic biosynthesis of cholesterol via increased SREBP-2 activity as well as cholesterol efflux by upregulating ATP-binding cassette transporter-A1 (ABCA1) mRNA, effects similar to treatment with olive oil phenols (Lammi et al., 2020). In mice treated with statins, cholesterol synthesis and total body cholesterol increase robustly, while plasma cholesterol decreases (Schonewille et al., 2016). Perhaps elevated maternal serum sFlts may be viewed as a sign of reduced accessible membrane cholesterol and increased cholesterol synthesis.

Low oxygen conditions such as high altitude and early first-trimester pregnancy increase sFlt-1 expression in placental villi, effects mediated by hypoxia-inducible factor-1 (Nevo et al., 2006). In preeclampsia, both increased membrane cholesterol in rafts and stiffer membranes due to increased cytoskeletal elements might slow transmembrane oxygen diffusion. In passing from maternal lungs to fetal blood, oxygen must diffuse across many cells and membranes: maternal alveolar cells, maternal endothelial cells, maternal red blood cells, placental trophoblasts, fetal endothelial cells, and finally fetal red blood cells. With the diffusion rate slowed at each transmembrane passage, cellular hypoxia may become clinically relevant. Thus, lipid variance may be the source of increased HIF signaling and sFlts seen in preeclampsia (Subczynski et al., 2021).

## 9 Lipidomics of preeclampsia

Fetal growth is dependent on a flow of nutrients from the maternal circulation, while excess fetal metabolites such as carbon dioxide, oxysterols, or fatty acids may either be transferred across the placenta to the maternal circulation or deposited in the placenta. Lipidomic analyses in pregnancy may evaluate maternal, placental, or fetal compartments. The balance between maternal and fetal metabolism and the enormous diversity of the human lipidome add complexity while discerning meaningful patterns in preeclampsia lipidomics.

Teleologically, the goal of the fetal/maternal/placental unit is to create a viable fetus and healthy mother with a disposable placenta. Within this framework, the placenta perhaps represents a depository for waste molecules before ultimate disposal. This may explain the findings in preeclampsia of accumulation of TUFAs in third-trimester placentas (Wada et al., 2017); significantly increased highly polyunsaturated cholesterol esters, triacylglycerols, and phosphatidylcholines; increased total phospholipids (Brown et al., 2016); and increased sphingomyelin (Del Gaudio et al., 2020). When compared to controls, preeclampsia maternal serum has increased PUFAs (Al et al., 1995; Anand et al., 2016; Irwinda et al., 2021) with an elevated omega 6:3 ratio (Williams et al., 1995) and increased oxidation of several lipids (He et al., 2021; Mauro et al., 2022), while lipids are relatively normal in fetal blood (van der Schouw et al., 1991). The finding of increased *de novo* synthesis of ceramides in the placenta, increased maternal serum ceramides, and reduced lysosomal breakdown prompted one research group to typify preeclampsia as a sphingolipid storage disease (Melland-Smith et al., 2015). Compared to controls, in preeclampsia, phosphatidylserine, which is normally found only on the inner leaflet of cell membranes, is markedly increased in maternal plasma (Korkes et al., 2014; Wojcik-Baszko et al., 2018) and in syncytiotrophoblastic microvesicles (Baig et al., 2013), membrane structures which are shed from the placenta into maternal circulation. As mentioned previously, ceramides increase transmembrane flip-flop, which may explain the increase in phosphatidylserines.

Summarizing the lipidomics of maternal blood in preeclampsia, in comparison to controls, increases are seen in both high melting point lipids such as saturated sphingomyelin and long-chain saturated fatty acids, and in low melting point phospholipids such as phosphatidylcholines, phosphatidylserines, and phosphatidylethanolamine with polyunsaturated and short fatty acyl groups. As a class, phosphatidylserines are increased, and phosphatidylinositols are markedly decreased (Odenkirk et al., 2020). There is an increased unsaturation index in maternal blood with an elevated omega 6:3 ratio and increased oxidized phospholipids (He et al., 2021), despite increased antioxidants such as flavonoids (Korkes et al., 2014). In preeclampsia, the placenta contains increased sphingomyelins, ceramides, and neutral lipids such as highly unsaturated cholesterol esters, triacylglycerols, and phospholipids. Phosphatidylinositols, which are known to regulate intracellular cholesterol transport (Hu et al., 2018), are decreased in maternal serum compared to controls. Compared to controls, phosphatidylethanolamine 37:2 is significantly increased (He et al., 2021).

### 9.1 Excess membrane rafts and preeclampsia lipidomics

In responding to a diet that contains excessive PUFAs, cells must not only increase high melting point lipids to regain membrane homeoviscosity but also remove oxidized lipids damaged by excessive free radical propagation. What is the evidence that lipidomic changes in preeclampsia are consistent with these metabolic goals? The overall increased unsaturation index of fatty acids in maternal serum and placenta is consistent with the known dietary changes of the last century, as is the elevated omega 6:3 fatty acid ratio. Increases in high melting point lipids, as seen in some lipidomic data sets, are expected in order to maintain membrane homeoviscosity. An alternate method of providing lipid bilayer homeoviscosity in an environment of high PUFAs would be to substitute a phospholipid head group such as phosphatidylethanolamine, which reduces membrane fluidity in comparison to its phosphatidylcholine analogs, perhaps explaining the significant increase in phosphatidylethanolamine 37:2 seen in He’s data. Lipidomic analysis of syncytiotrophoblastic microvesicles, which are shed from placenta membranes, shows a unique reversal of the SM-to-phosphatidylcholine (PC) ratio with an SM/PC of 3:1 in preeclampsia versus 1:3 in controls (Baig et al., 2013), perhaps indicating increased membrane rafts in preeclampsia, as is known to occur in cardiovascular diseases. One study shows increased placental plasmalogens (Brien et al., 2017), a type of phospholipid that protects unsaturated fatty acids from oxidation. With limited accessible membrane cholesterol, phosphatidylinositol, which stimulates reverse cholesterol transport (Burgess et al., 2003), is downregulated as reported.

This interpretation of lipidomic data is supported by other pathological findings in preeclampsia, which indicate reduced accessible membrane cholesterol. Increased activity of sterol regulatory element binding protein1-c (SREBP) transcription factors, which stimulate sterol synthesis when cellular cholesterol levels are low, is found in maternal serum and placental tissue in preeclampsia (Vásárhelyi et al., 2006; Jianhua et al., 2016), an indication of increased demand for cholesterol. Increased ceramides in preeclampsia, which can displace cholesterol from sphingomyelin to form accessible cholesterol, also indicate reduced accessible membrane cholesterol. These findings are consistent with responses triggered by a diet with excess PUFAs and a high omega 6:3 ratio, increased TUFAs, and reduced accessible membrane cholesterol.

## 10 Discussion

In preeclampsia, the paradox of cholesterol-dependent processes such as Wnt/catenin signaling, Hh signaling, and endothelial nitric oxide synthesis being downregulated with increased systemic cholesterol and cell membrane cholesterol is understandable if we consider the potential effects of lipid variance in modern diets. Lipidomic analyses of maternal, placental, and fetal tissues characterize preeclampsia as a disease with a high fatty acid unsaturation index and markedly increased lipid oxidation, as expected with a high dietary PUFA. Elevated placental TUFAs are consistent with increased dietary intake. The increased cellular stiffness of preeclampsia is expected when PUFAs are incorporated into membrane lipid bilayers. Increased melting point disparity in maternal endothelial membranes increases cholesterol sequestration, as do elevated TUFAs and cholesterol esterification with oxidized fatty acids. As a result of cholesterol serving as an antioxidant, oxysterols are increased. The transplacental movement of cholesterol to the fetus also reduces accessible cholesterol. In this way, the paradox of increased systemic cholesterol with reduced accessible cholesterol may be explained. Thus, dietary lipid variance may explain dyslipidemia, abnormal raft formation and signaling, excessive oxidative stress, inflammation, ceramides, and thromboses that characterize preeclampsia. Reduced accessible cholesterol impairs Hh and Wnt/catenin signaling, which are crucial in normal placental development. Limited accessible cholesterol and increased cytoskeletal elements are also likely to cause the reduced and shortened cilia of preeclampsia.

Some findings in human lipidomics and this theoretical model differ from results in experimental animal models of preeclampsia. A common murine model uses reduced uterine artery perfusion pressure to mimic the shallow trophoblast invasion of uterine spiral arteries, thereby producing progressive placental ischemia. This model has been successful in reproducing hypertension, oxidative stress, fetal growth restriction, and other clinical findings of preeclampsia and has contributed significantly to understanding preeclampsia pathology. Combining this model with super physiologic concentrations of dietary cholesterol, a model of severe preeclampsia has been created (Johnson et al., 2014; Sáez et al., 2020). Initially, these models seem to contradict the reasoning that excessive PUFAs and reduced accessible membrane cholesterol are the sources of oxidative stress and poor placentation in preeclampsia. However, the fact that rats, unlike humans, lack cholesteryl ester transfer activity (CETP) (Oschry and Eisenberg, 1982; Hogarth et al., 2003), but have robust lecithin cholesterol acyl transferase (LCAT) activity, means that they are unable to offload cholesterol esters from HDL to LDL and VLDL and must rely on a very efficient HDL reflux system to remove excessive cholesterol. At the super-physiologic concentrations of cholesterol used in these experiments, this system may be overwhelmed. A normal rodent diet contains 10% fat, while the typical American or European diet contains 36%–40% fat (Speakman, 2019) with an average cholesterol intake of 293 mg (2.637 kcal) or about 1/1,000 of daily dietary calories supplied as cholesterol in humans. A high-cholesterol laboratory diet in rodents provides 1%–2% of daily calories as cholesterol, a level one to two thousand times greater than the average human cholesterol intake as a percentage of daily calories in an animal with a lower baseline fat intake. In addition, due to higher metabolic rates, rodents have a higher tissue requirement for omega-3 fatty acids, impairing translational validity between species (Whelan and Whelan, 2020).

This model showing the potential role of dietary lipids in preeclampsia pathology raises the possibility of treatment using inexpensive dietary lipids. Because fatty acids are rapidly (within hours) incorporated into the endothelium after absorption from the gastrointestinal tract (Levental, 2020), supplementing lipids to correct dysfunction in signaling from cilia might reasonably be expected to correct the acute pathological dysfunctions of the maternal endothelium found in early-onset severe preeclampsia within a clinically relevant time frame. The rapid resolution of preeclampsia postpartum is also reassuring regarding the time frame of treatment. A recommended treatment might include supplementing medium-chain saturated (coconut oil) and monounsaturated fatty acids (olive oil) to displace damaged fatty acids and maintain optimal maternal endothelial viscosity, eliminating dietary TUFAs, reducing dietary omega-6 PUFAs and supplementing with omega-3 fatty acids, and supplementing with medium and monounsaturated cholesterol esters to allow rapid transesterification and removal of damaged fatty acids and oxysterols from the maternal endothelium.

The use of lipids to treat metabolic diseases is not new. The interest of the author in treating preeclampsia with dietary lipids arose after witnessing the rapid resolution of early severe preeclampsia at 27 weeks gestational age after a change in the maternal diet. Recent studies using olive oil to treat conditions including burns (Ghanbari et al., 2023), diabetes (Schwingshackl et al., 2017), chronic kidney disease (Romani et al., 2020), and a mouse model of Alzheimer’s disease (Abdallah et al., 2023) all show beneficial effects. In a group of 800 pregnant women, a randomized, controlled trial of the reduction of dietary TUFAs showed a significant difference in TUFA intake between study groups, which was accompanied by a hazard ratio for preeclampsia reduced to 0.56 in the treated group (Alamolhoda et al., 2020). As anticipated, studies supplementing omega-3 PUFA, a very low melting point lipid, have not shown significant benefit (Zhou et al., 2012; Middleton et al., 2018), although theoretically, a reduction of the omega 6:3 ratio to less than 4:1 should reduce inflammation and thromboses. These studies did not assess efficacy in reducing maternal omega 6:3 ratios. A systematic review of the effects of olive oil consumption on maternal–fetal outcomes shows that higher olive oil consumption is associated with a lower risk of gestational diabetes, preeclampsia, and cardiovascular risk (Cortez-Ribeiro et al., 2023), and patients who adhered most closely to a Mediterranean diet pattern had a significantly lower risk of preeclampsia (Makarem et al., 2022). These studies justify cautious optimism that acquired ciliopathies, including preeclampsia, may also respond to dietary lipid therapy.

## Data Availability

The original contributions presented in the study are included in the article/Supplementary Material; further inquiries can be directed to the corresponding author.
